# Genome features of moderately halophilic polyhydroxyalkanoate-producing *Yangia* sp. CCB-MM3

**DOI:** 10.1186/s40793-017-0232-8

**Published:** 2017-01-23

**Authors:** Nyok-Sean Lau, Ka-Kei Sam, Abdullah Al-Ashraf Amirul

**Affiliations:** 1 Centre for Chemical Biology, Universiti Sains Malaysia, Bayan Lepas, 11900 Penang Malaysia; 2 School of Biological Sciences, Universiti Sains Malaysia, Minden, 11800 Penang Malaysia

**Keywords:** *Yangia*, *Rhodobacteraceae*, Matang mangrove, Halophile, Polyhydroxyalkanoate

## Abstract

*Yangia* sp. CCB-MM3 was one of several halophilic bacteria isolated from soil sediment in the estuarine Matang Mangrove, Malaysia. So far, no member from the genus *Yangia*, a member of the *Rhodobacteraceae* family, has been reported sequenced. In the current study, we present the first complete genome sequence of *Yangia* sp. strain CCB-MM3. The genome includes two chromosomes and five plasmids with a total length of 5,522,061 bp and an average GC content of 65%. Since a different strain of *Yangia* sp. (ND199) was reported to produce a polyhydroxyalkanoate copolymer, the ability for this production was tested *in vitro* and confirmed for strain CCB-MM3. Analysis of its genome sequence confirmed presence of a pathway for production of propionyl-CoA and gene cluster for PHA production in the sequenced strain. The genome sequence described will be a useful resource for understanding the physiology and metabolic potential of *Yangia* as well as for comparative genomic analysis with other *Rhodobacteraceae*.

## Introduction

*Yangia* is a genus of the *Roseobacter* group, within the family *Rhodobacteraceae*, order *Rhodobacterales*, class *Alphaproteobacteria*, thus far containing only one species *Yangia pacifica* [1, 2]. Members of the *Roseobacter* clade have been widely detected in marine environments, from coastal to open ocean and from surface of the water to abyssal depths [3]. The type strain of *Y. pacifica*, DX5-10^T^ was isolated from coastal sediment of the East China Sea of the Pacific Ocean [1]. The accumulation of poly(3-hydroxybutyrate), P(3HB) in *Y. pacifica* DX5-10 was observed. *Yangia* sp. strain ND199 was recently reported to produce poly(3-hydroxybutyrate-*co*-3-hydroxyvalerate), P(3HB-*co*-3HV) from structurally unrelated carbon sources [4]. So far, only few bacteria including *Haloferax mediterranei*, ‘*Nocardia* *corallinia’*, *Pseudomonas* sp. EL-2, *Rhodococcus* sp. NCIMB 40126 and recombinant *Escherichia coli* can synthesize P(3HB-*co*-3HV) from single unrelated carbon sources [5–9]. The incorporation of 3HV into 3HB-based polymer increases the flexibility, impact resistance as well as ductility of the polymer [10] and makes the polymer suitable for many industrial applications.

Mangroves are highly productive ecosystems covering approximately 75% of the total tropical and subtropical coastlines. Apart from wood production, mangrove forests support a wide range of functions including coastline protection, nutrient cycling, habitat for endangered species, breeding ground for marine life and have been proven as natural barrier againt tsunami [11]. Matang mangrove, Malaysia is widely regarded as the best-managed sustainable mangrove ecosystem in the world. *Yangia* sp. CCB-MM3, analyzed in the present study, was isolated from soil samples obtained from the Matang mangrove. The sampling location was situated in estuarine mangrove ecosystem that is under both the influence of marine condition and the flow of freshwater. Saline environments including estuaries and coastal marine sites have been focus of study for halophilic organisms that flourish in these habitats. Halophiles have attracted interest as candidates for bioprocessing because of their unique property including the ability to grow in high salt containing media, allowing fermentation processes to run contamination free under non-sterile condition [12].

At the time of writing, there are more than 300 genome assemblies from members of the family *Rhodobacteraceae* but the complete genome from the genus *Yangia* has not been reported. Here, we present the first complete genome of a *Yangia* representative and insight into the genes or pathways for polyhydroxyalkanoate (PHA) biosynthesis in this halophilic bacterium.

## Organism information

### Classification and features

**Fig. 1:**
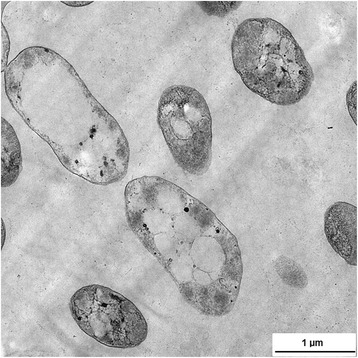
Transmission electron micrograph of *Yangia* sp. CCB-MM3 cells containing PHA granules

**Fig. 2:**
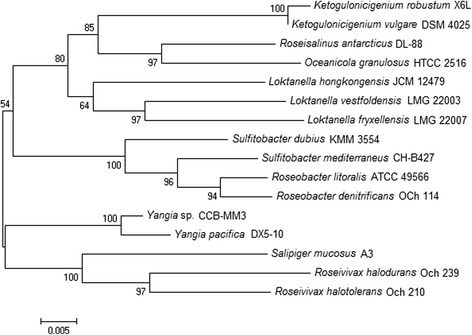
Phylogenetic tree highlighting the position of *Yangia* sp. strain CCB-MM3 relative to other strains within the *Rhodobacteraceae* family. The phylogenetic tree was constructed based on 16S rRNA gene sequences using neighbour-joining method [42] with Kimura two-parameter model derived from MEGA6 [43]

Soil sediment samples (0–10 cm) were collected from Matang Mangrove (4.85228 N, 100.55777 E) located on the west coast of Penisular Malaysia in October 2014 [13]. The soil samples had moderate salinity (21 ppt) and the temperature was 30 °C on the day of sampling. CCB-MM3 was isolated from the soil samples on low nutrient artificial seawater medium (L-ASWM) agar plates [14]. Bacteriological characteristics of the isolate are summarized in Table 1. The isolate is a Gram-negative, motile and rod-shaped bacterium of 1–2 μm in size (Fig. 1). The strain exhibited growth at 20–40 °C (optimum 30 °C) and pH 5–10 (optimum pH 7.5). Transmission electron microscopy revealed the presence of discrete, electron-transparent inclusions in the cytoplasm of strain CCB-MM3, presumably containing accumulated PHA granules. There are five identical 16S rRNA gene copies in CCB-MM3 genome. When compared to the 16S prokaryotic rRNA database available at EzTaxon [15], the 16S rRNA gene sequence of CCB-MM3 exhibited an identity of 98.8% with the type strain *Y. pacifica* DX5-10. A phylogenetic tree was constructed on the basis of 16S rRNA gene sequences of strain CCB-MM3 and other members of the family *Rhodobacteraceae*. The 16 s rRNA gene sequence phylogeny placed CCB-MM3 in the same cluster as *Y. pacifica* DX5-10 (Fig. 2). The high 16S rRNA gene sequence similarity and distinct phylogenetic lineage with *Y. pacifica* DX5-10 suggest that the strain CCB-MM3 belongs to the genus *Yangia*.

**Table 1:** Classification and general features of *Yangia* sp. strain CCB-MM3

MIGS ID	Property	Term	Evidence code^a^
	Classification	Domain *Bacteria*	TAS [36]
		Phylum *Proteobacteria*	TAS [37]
		Class *Alphaproteobacteria*	TAS [38]
		Order *Rhodobacterales*	TAS [39]
		Family *Rhodobacteraceae*	TAS [40]
		Genus *Yangia*	TAS [1]
		Species *Yangia* sp.	
		Strain CCB-MM3	
	Gram stain	Negative	IDA
	Cell shape	Rod	IDA
	Motility	Motile	IDA
	Sporulation	Non-sporulating	NAS [1]
	Temperature range	20–40 °C	IDA
	Optimum temperature	30 °C	IDA
	pH range; Optimum	5–10; 7.5	IDA
	Carbon source	Maltose, lactate, malate, arginine, glutamate	NAS [1]
MIGS-6	Habitat	Environment	IDA
MIGS-6.3	Salinity	1–10%	IDA
MIGS-22	Oxygen requirement	Aerobic	NAS [1]
MIGS-15	Biotic relationship	Free-living	NAS
MIGS-14	Pathogenecity	Non-pathogenic	NAS
MIGS-4	Geographic location	Malaysia	IDA
MIGS-5	Sample collection	October 2014	IDA
MIGS-4.1	Latitude	4.85228 N	IDA
MIGS-4.2	Longitude	100.55777 E	IDA
MIGS-4.4	Altitude	Sea level	IDA

^a^Evidence codes - IDA: Inferred from Direct Assay; TAS: Traceable Author Statement (i.e., a direct report exists in the literature); NAS: Non-traceable Author Statement (i.e., not directly observed for the living, isolated sample, but based on a generally accepted property for the species, or anecdotal evidence). These evidence codes are from the Gene Ontology project [41]

## Genome sequencing information

### Genome project history

*Yangia* sp. CCB-MM3 was selected for genome sequencing on the basis of its physiological and phenotypical features, and was part of a study aiming at characterizing the microbiome of mangrove sediments. Genome assembly and annotation were performed at the Centre for Chemical Biology, Universiti Sains Malaysia. The genome project was deposited at GenBank under the accession PRJNA310305. Table 2 summarizes the project information in accordance with the Minimum Information about a Genome Sequence (MIGS).

**Table 2:** Genome sequencing project information

MIGS ID	Property	Term
MIGS-31	Finishing quality	Finished
MIGS-28	Libraries used	PacBio SMRTbell 10 Kb library
MIGS-29	Sequencing platforms	PacBio RS II
MIGS-31.2	Fold coverage	300 x
MIGS-30	Assemblers	HGAP2
MIGS-32	Gene calling method	RAST
	Locus tag	AYJ57
	GenBank ID	CP014595-CP014601
	GenBank date of release	July 18, 2016
	GOLD ID	Gp0155985
	BIOPROJECT	PRJNA310305
MIGS-13	Source material identifier	CCB-MM3
	Project relevance	Biotechnology, environmental

### Growth conditions and genomic DNA preparation

*Yangia* sp. CCB-MM3 cells for genome sequencing was grown in L-ASWM [0.05% tryptone, 2.4% (*w*/*v*) artificial sea water mix (Marine Enterprises International, USA), pH 7.6] under rotation at 30 °C [14]. Genomic DNA extraction was performed using the DNeasy Blood and Tissue Kit (Qiagen, USA). The genomic DNA was quantified using Qubit 3.0 Fluorimeter (Life Technologies, USA) and visualized by agarose gel electrophoresis (0.7%).

To promote PHA biosynthesis in *Yangia* sp. CCB-MM3, one-stage cultivation was carried out. Pre-culture of strain CCB-MM3 was prepared by growing cells on moderate halophiles (HM) medium containing per litre: 45 g NaCl, 0.25 g MgSO_4_^.^7H_2_O, 0.09 g CaCl_2_.2H_2_O, 0.5 g KCl, 0.06 g NaBr, 5 g peptone, 10 g yeast extract and 1 g glucose at 30 °C with rotary shaking at 200 rpm for 6 h. Subsequently, 3% (v/v) inoculum (OD_600nm_ = 4) was transferred into HM-1 medium containing per litre: 45 g NaCl, 0.25 g MgSO_4_.7H_2_O, 0.09 g CaCl_2_.2H_2_O, 0.5 g KCl, 0.06 g NaBr, 0.25 g KH_2_PO_4_, 2 g yeast extract and 20 g glycerol [4]. The culture was incubated at 30 °C, 200 rpm for 48 h before being harvested. PHA was extracted from lyophilized cells according to the method described previously [16]. ^1^H nuclear magnetic resonance spectrum was obtained in deuterated chloroform solution of the PHA polymer (25 mg/mL) recorded on a Bruker spectrometer (Bruker, Switzerland) at frequency of 400 MHz.

### Genome sequencing and assembly

Whole genome sequencing of *Yangia* sp. CCB-MM3 was performed using the PacBio technology. In short, a library was prepared following the PacBio 10 Kb SMRTbell library preparation protocol. The final library was size selected using Blue Pippin electrophoresis (Saga Science, USA). The library was sequenced using two SMRT cells on PacBio RS II platform using P6-C4 chemistry. The run generated 153,311 reads with an average length of 14.46 Kb and a total of 2.22 Gb data. Raw reads were filtered and *de novo* assembled using hierarchical genome-assembly process v2 protocol in SMRT Analysis v2.3.0 [17]. Two rounds of genome polishing were performed using Quiver to improve the accuracy of the assembly.

### Genome annotation

The genome annotation was performed using the rapid annotation using subsystem technology [18]. The predicted *Yangia* sp. protein sequences were compared against the clusters of orthologous groups database using BLASTP. Non-coding genes and miscellaneous features were predicted using tRNAscan-SE [19], SignalP [20], TMHMM [21] and CRISPRFinder [22].

## Genome properties

**Fig. 3:**
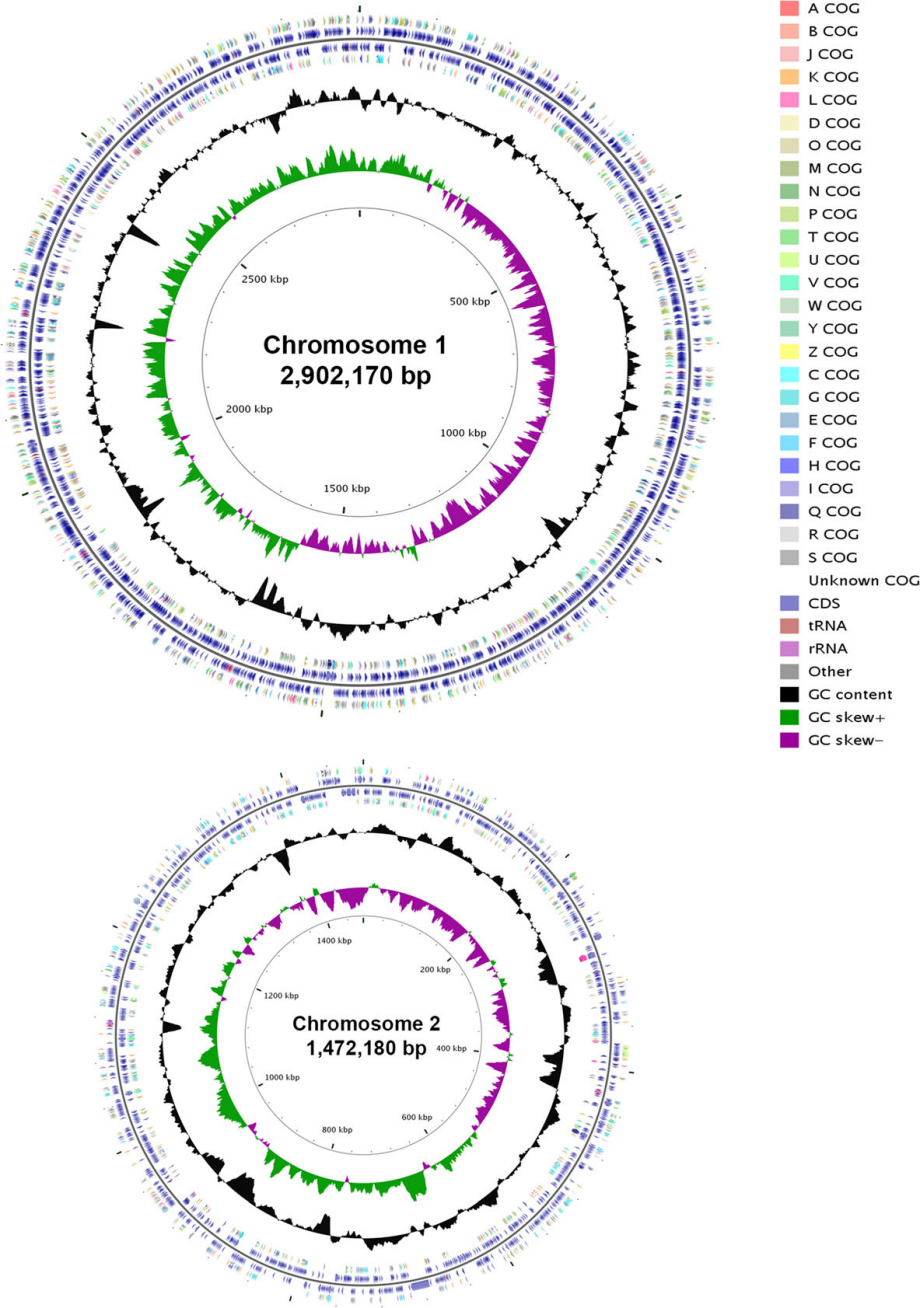
Graphical map showing only chromosomes of *Yangia* sp. CCB-MM3 generated with CGview comparison tool [44]. From outside to the center: genes identified by the COG on forward strand, CDS on forward strand, CDS on reverse strand, genes identified by the COG on reverse strand, RNA genes (tRNAs orange, rRNAs pink, other RNAs grey), GC content (black) and GC skew (purple/green)

The genome of *Yangia* sp. CCB-MM3 is 5,522,061 bp-long and consists of two circular chromosomes and five plasmids (Table 3 and Fig. 3). The genome has a 64.98% GC content (Table 4). There are 5027 predicted protein-coding genes and 69 RNA genes (five rRNA operon and 44 tRNAs). 49 RNA genes are found on chromosome 1 while 20 are on chromosome 2. Of the predicted protein-coding genes, 3774 were assigned with a putative function, while the remaining were annotated as hypothetical proteins. A total of 3945 genes were assigned to COG categories (2343 on chromosome 1; 1068 on chromosome 2; the remaining on plamids) and a breakdown of their functional assignments is shown in Table 5. The most abundant COG functional category in strain CCB-MM3 were amino acid transport and metabolism, general function prediction only and carbohydrate transport and metabolism.

**Table 3:** Genome composition for *Yangia* sp. CCB-MM3

Label	Size (Mb)	Topology	INSDC identifier	RefSeq ID
Chromosome 1	2.902	circular	CP014595	NZ_CP014595.1
Chromosome 2	1.472	circular	CP014596	NZ_CP014596.1
Plasmid 1	0.316	circular	CP014597	NZ_CP014597.1
Plasmid 2	0.274	circular	CP014598	NZ_CP014598.1
Plasmid 3	0.281	circular	CP014599	NZ_CP014599.1
Plasmid 4	0.223	circular	CP014600	NZ_CP014600.1
Plasmid 5	0.054	circular	CP014601	NZ_CP014601.1

**Table 4:** Genome statistics

Attribute	Value	% of total
Genome size (bp)	5,522,061	100.00
DNA coding (bp)	4,744,053	85.91
DNA G + C (bp)	3,588,235	64.98
DNA scaffolds	7	100.00
Total genes	5096	100.00
Protein coding genes	5027	98.65
RNA genes	69	1.35
Pseudo genes	61	1.20
Genes in internal clusters	NA	NA
Genes with function prediction	3774	74.06
Genes assigned to COGs	3945	77.41
Genes with Pfam domains	4244	83.28
Genes with signal peptides	461	9.05
Genes with transmembrane helices	1123	22.04
CRISPR repeats	2	0.04

**Table 5:** Number of genes associated with general COG functional categories

Code	Value	% age	Description
J	189	3.76	Translation, ribosomal structure and biogenesis
A	0	0.00	RNA processing and modification
K	350	6.96	Transcription
L	190	3.78	Replication, recombination and repair
B	3	0.06	Chromatin structure and dynamics
D	33	0.66	Cell cycle control, cell division, chromosome partitioning
V	45	0.90	Defense mechanisms
T	153	3.04	Signal transduction mechanisms
M	252	5.01	Cell wall/membrane biogenesis
N	49	0.97	Cell motility
U	55	1.09	Intracellular trafficking and secretion
O	139	2.77	Posttranslational modification, protein turnover, chaperones
C	276	5.49	Energy production and conversion
G	374	7.44	Carbohydrate transport and metabolism
E	615	12.23	Amino acid transport and metabolism
F	107	2.13	Nucleotide transport and metabolism
H	163	3.24	Coenzyme transport and metabolism
I	169	3.36	Lipid transport and metabolism
P	288	5.73	Inorganic ion transport and metabolism
Q	176	3.50	Secondary metabolites biosynthesis, transport and catabolism
R	582	11.58	General function prediction only
S	348	6.92	Function unknown
–	1082	21.52	Not in COGs

## Insights from the genome sequence

*Yangia* sp. CCB-MM3 has a large repertoire of genes involved in central carbon metabolism. Briefly, central carbon metabolism in CCB-MM3 includes a complete set of genes encoding glycolysis/gluconeogenesis, pentose phosphate pathway and tricarboxylic acid cycle. *Yangia* sp. CCB-MM3 was isolated from mangrove soil, one of the most carbon-rich ecosystems. Therefore, it is no surprise that the genome of CCB-MM3 comprised a considerable number of carbohydrate-active enzymes including 71 glycosyl transferases, 50 glycoside hydrolases (GH), 31 carbohydrate binding modules and 23 carbohydrate esterases (Table 6). CCB-MM3 contains genes representing 19 GH families (GH 1, 4, 8, 13, 16, 23, 25, 28, 30, 39, 51, 74, 77, 102, 103, 104, 105, 108 and 109) and some of these genes are involved in the utilization of saccharides including _D_-galacturonate, _D_-glucoronate, sucrose, maltose, maltodextrin and glycogen (Table 7).

**Table 6:** Carbohydrate active enzymes (CAZy) in the genome of *Yangia* sp. CCB-MM3

Glycoside hydrolase	No. of genes	Glycosyl transferase	No. of genes	Carbohydrate binding module	No. of genes	Carbohydrate esterase	No. of genes
GH1	1	GT2	22	CBM6	3	CE1	8
GH4	1	GT4	22	CBM14	1	CE3	1
GH8	1	GT5	1	CBM35	9	CE4	7
GH13	9	GT8	1	CBM44	2	CE9	1
GH16	2	GT14	2	CBM48	7	CE10	3
GH23	8	GT19	1	CBM50	4	CE11	1
GH25	1	GT20	1	CBM57	5	CE14	1
GH28	1	GT21	2			CE16	1
GH30	1	GT26	4				
GH39	2	GT28	1				
GH51	3	GT30	2				
GH74	1	GT35	1				
GH77	1	GT51	3				
GH102	1	GT81	1				
GH103	5	GT83	1				
GH104	1	GT89	3				
GH105	2	GT92	3				
GH108	1						
GH109	8

**Table 7:** Glycoside hydrolase genes in the genome of *Yangia* sp. CCB-MM3

GH family	Annotation	Locus tag
GH1	Beta-galactosidase	AYJ57_00695
GH4	_L_-Lactate dehydrogenase	AYJ57_06470
GH8	Hypothetical protein	AYJ57_03365
GH13	Glycogen debranching enzyme	AYJ57_00665
	Glycogen-branching enzyme	AYJ57_00680
	Alpha-glucosidase	AYJ57_00720
	Glycogen-branching enzyme	AYJ57_09210
	Hypothetical protein	AYJ57_09215
	Alpha-amylase	AYJ57_12455
	Malto-oligosyltrehalose synthase	AYJ57_24365
	Malto-oligosyltrehalose trehalohydrolase	AYJ57_24370
	Glycogen debranching enzyme	AYJ57_24375
GH16	Hypothetical protein	AYJ57_23180
	Hypothetical protein	AYJ57_23220
GH23	Lytic transglycosylase	AYJ57_02155
	Lytic transglycosylase	AYJ57_04690
	Lytic transglycosylase	AYJ57_06695
	Transglycosylase	AYJ57_11460
	Lytic murein transglycosylase	AYJ57_15595
	Tail length tape measure protein	AYJ57_16590
	Hypothetical protein	AYJ57_22680
	Transglycosylase	AYJ57_12770
GH25	Glycoside hydrolase	AYJ57_19400
GH28	Polygalacturonase	AYJ57_18585
GH30	Hypothetical protein	AYJ57_13245
GH39	Hypothetical protein	AYJ57_22570
	Hypothetical protein	AYJ57_22600
GH51	Hypothetical protein	AYJ57_22330
	Type I secretion protein	AYJ57_21970
	Type I secretion protein	AYJ57_23060
GH74	Glycoside hydrolase	AYJ57_16805
GH77	4-Alpha-glucanotransferase	AYJ57_00660
GH102	Murein transglycosylase	AYJ57_07750
GH103	Lytic transglycosylase	AYJ57_08665
	Murein transglycosylase	AYJ57_13070
	Murein transglycosylase	AYJ57_05515
	Murein transglycosylase	AYJ57_06735
	Hypothetical protein	AYJ57_22810
GH104	Hypothetical protein	AYJ57_21640
GH105	Di-trans,poly-cis-decaprenylcistransferase	AYJ57_18580
	Glycosyl hydrolase family 88	AYJ57_21240
GH108	Peptidoglycan-binding protein	AYJ57_00570
GH109	Oxidoreductase	AYJ57_07230
	Oxidoreductase	AYJ57_10590
	Oxidoreductase	AYJ57_11790
	Galactose 1-dehydrogenase	AYJ57_16180
	Oxidoreductase	AYJ57_20060
	Oxidoreductase	AYJ57_20220
	Inositol 2-dehydrogenase	AYJ57_20225
	Oxidoreductase	AYJ57_23310

Some species from the *Roseobacter* clade have been characterized as essential players in biogeocycling of organic or inorganic sulfur-containing compounds [23–25]. The genome of *Yangia* sp. CCB-MM3 encodes the enzymes necessary for assimilatory sulfate reduction including sulfate adenyltransferase (AYJ57_25280), adenylnylsulfate kinase (AYJ57_25275), phosphoadenylylsulfate reductase (AYJ57_02835) and sulfite reductase (AYJ57_02830). Interestingly, CCB-MM3 genome also harbours the complete set of sulfur-oxidizing genes including *soxX* (AYJ57_01935), *soxY* (AYJ57_01940), *soxZ* (AYJ57_01945), *soxA* (AYJ57_01950), *soxB* (AYJ57_01955), *soxC* (AYJ57_01960) and *soxD* (AYJ57_01965) for thiosulfate oxidation *in vitro*. SoxYZ is the carrier protein that interacts with SoxAX, SoxB and SoxCD; SoxAX cytochrome complex is proposed to link sulfur substrate to SoxYZ; dimanganese SoxB removes oxidized sulfur residue from SoxYZ through hydrolysis; and SoxCD catalyzes the oxidation of reduced sulfur residue bound to SoxYZ [26–29]. These genes encoding essential components of the Sox multienzyme complex are organized in a single locus in CCB-MM3. Analysis of *Yangia* sp. CCB-MM3 genome also revealed that rodanese-like sulfurtransferases (AYJ57_05465, AYJ57_08495, AYJ57_10220, AYJ57_16970 and AYJ57_24415) that can participate in the metabolism of thiosulfate and elemental sulfur during disproportionation are present in the genome.

Although the ability of *Yangia* to grow with free nitrogen gas as sole nitrogen source has not been analyzed yet, all genes necessary for nitrogen fixation were identified in the genome of *Yangia* sp. CCB-MM3. The genome encodes the subunits α and β of molybdenum-iron nitrogenase (AYJ57_00195, AYJ57_00200), its regulatory and accessory proteins (AYJ57_00310, AYJ57_00210, AYJ57_00215 and AYJ57_00315).

### PHA metabolism

**Fig. 4:**
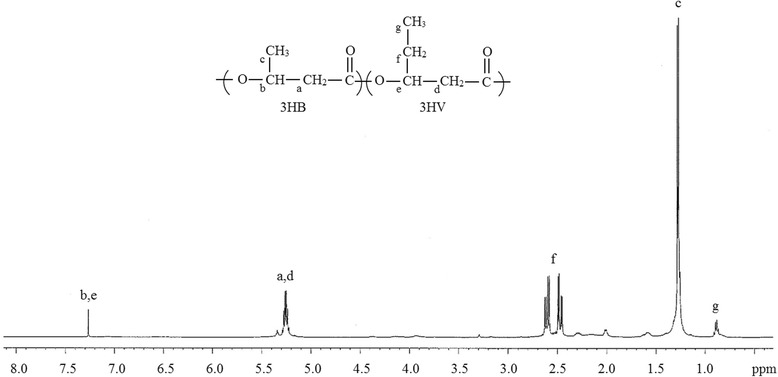
^1^H-NMR spectrum of P(3HB-*co*-3HV) isolated from *Yangia* sp. CCB-MM3 grown on glycerol

The ability of *Yangia* sp. CCB-MM3 to accumulate the copolymer P(3HB-*co*-3HV) with 7 mol% of 3HV from structurally unrelated carbon source was confirmed by NMR analysis (Fig. 4). In *‘Norcadia corallina’* and *Rhodococcus ruber*, P(3HB-*co*-3HV) is synthesized from simple carbon source by using a pathway in which majority of propionyl-CoA is derived from the methylmalonyl-CoA pathway [30]. Similarly, genes encoding for complete methylmalonyl-CoA pathway were identified in *Yangia* sp. CCB-MM3 (Table 8), suggesting that this is one of the potential pathways involved in providing propionyl-CoA in *Yangia* sp. Succinyl-CoA is an important intermediate of the methylmalonyl-CoA pathway. The isomerization of succinyl-CoA to (*R*)-methylmalonyl-CoA proceeds through the action of methylmalonyl-CoA mutase (AYJ57_16720). (*R*)-methylmalonyl-CoA is converted to the (*S*) form via methylmalonyl-CoA epimerase (AYJ57_06825). The latter is then decarboxylated to propionyl-CoA by methylmalonyl-CoA decarboxylase (AYJ57_16710).

**Table 8:** Genes involved in PHA metabolism in *Yangia* sp. CCB-MM3

Function	Gene	EC number	No. of genes
Propionyl-CoA supplying pathway			
Methylmalonyl-CoA mutase	*mcm*	5.4.99.2	1
Methylmalonyl-CoA epimerase	*mce*	5.1.99.1	1
Methylmalonyl-CoA decarboxylase	*mmcD*	4.1.1.41	1
PHA biosynthetic pathway			
β-ketothiolase	*phaA*	2.3.1.16	5
NADPH-dependent acetoacetyl-CoA reductase	*phaB*	1.1.1.36	3
PHA synthase	*phaC*	2.3.1.-	2
Other aspect of PHA metabolism			
PHA depolymerase	*phaZ*	3.1.1.75	2
Phasin	*phaP*	–	1
PHA synthesis regulator	*phaR*	–	1

The formation of P(3HB-*co*-3HV) from its precursors, acetyl-CoA and propionyl-CoA is catalyzed by three enzymes [10] and the genes encoding these enzymes were identified in the genome of CCB-MM3. The first reaction consists of either the condensation of two acetyl-CoA or condensation of acetyl-CoA and propionyl-CoA by β-ketothiolase encoded by multiple *phaA* in CCB-MM3 (AYJ57_07995, AYJ57_09725, AYJ57_11220, AYJ57_15015 and AYJ57_20090). The resulting intermediate is reduced to 3-hydroxybutyryl-CoA or 3-ketovaleryl-CoA by NADPH-dependent acetoacetyl-CoA reductase encoded by *phaB* (AYJ57_01725, AYJ57_11215 and AYJ57_24165). The hydroxyacyl-CoA monomers are then incorporated into the growing polymer chain by PHA synthase, encoded by *phaC* [31]. The genome of *Yangia* sp. CCB-MM3 possesses two PHA synthases genes, *phaC1*_*Ys*_ and *phaC2*_*Ys*_ (AYJ57_06535 and AYJ57_14600) that are located on chromosome 1 and 2, respectively. Both *phaC1*_*Ys*_ and *phaC2*_*Ys*_ encode 598 amino acid proteins which show 67 and 81% identity with *phaC* from *Citreicella* sp. SE45. These PHA synthases belong to Class I that have only one subunit and show preference to short chain length hydroxyacyl-CoA monomers [32].

Besides genes that are directly involved in PHA biosynthesis, gene involved in other aspect of PHA metabolism e.g. PHA depolymerase (*phaZ*) was annotated in the genome of *Yangia* sp. CCB-MM3. Since PHA is accumulated as storage compound for its producer, some PHA-producers harbour native machinery for the degradation of PHA. The synthesized PHA is catabolized by intracellular PhaZ and subsequently reutilized by cell [33]. However, mechanism of control for PHA biosynthesis or degradation in its native producer is not yet fully understood. Two PHA depolymerases, *phaZ1*_*Ys*_ and *phaZ2*_*Ys*_ (AYJ57_12275 and AYJ57_14595) were found in CCB-MM3. Another noncatalytic PHA granule-associated protein, phasin, was found to be encoded by single copy of *phaP* gene (AYJ57_14605) in CCB-MM3. Phasin has putative role in maintaining the stability of PHA granules formed by preventing the coalescence of separated granules [34]. The transcriptional repressor gene *phaR* (AYJ57_10595) that encodes for protein that regulates the transcription of *phaP* was also annotated in CCB-MM3 genome. It was proposed that PhaR functions as a repressor protein of transcription by binding to the upstream region of PhaP [35].

## Conclusions

At least 300 members of the family *Rhodobacteraceae* have publically accessible genomes. *Yangia* sp. CCB-MM3, however, represents the first sequenced genome from the genus. The strain was selected for genome sequencing by our research group as part of a study focusing on characterizing the microbiome of Malaysia mangrove sediments. The strain CCB-MM3 genome includes genes encoding monomer supplying and biosynthetic pathway for PHA production. Availability of the genome sequence will facilitate further study on the strain’s biological potential and provide reference material for comparative genomic analysis with other *Rhodobacteraceae*.

## Abbreviations

CBM: Carbohydrate binding module
CE: Carbohydrate esterase
COG: Clusters of orthologous groups
GH: Glycoside hydrolase
GT: Glycosyl transferase
HGAP: Hierarchical genome-assembly process
HM: Moderate halophiles medium
L-ASWM: Low nutrient artificial seawater medium
P(3HB): Poly(3-hydroxybutyrate)
P(3HB-*co*-3HV): Poly(3-hydroxybutyrate-*co*-3-hydroxyvalerate)
PHA: Polyhydroxyalkanoate
RAST: Rapid annotation using subsystem technology
SMRT: Single molecule real-time
